# Evaluation of a Secure Messaging System in the Care of Children With Medical Complexity: Mixed Methods Study

**DOI:** 10.2196/42881

**Published:** 2023-02-23

**Authors:** Camilla Parpia, Clara Moore, Madison Beatty, Susan Miranda, Sherri Adams, Jennifer Stinson, Arti Desai, Leah Bartlett, Erin Culbert, Eyal Cohen, Julia Orkin

**Affiliations:** 1 Temerty Faculty of Medicine University of Toronto Toronto, ON Canada; 2 SickKids Research Institute Toronto, ON Canada; 3 Division of Paediatric Medicine The Hospital for Sick Children Toronto, ON Canada; 4 Department of Anesthesia and Pain Medicine The Hospital for Sick Children Toronto, ON Canada; 5 Department of Pediatrics University of Washington Seattle, WA United States; 6 Royal Victoria Regional Health Center Barrie, ON Canada; 7 Credit Valley Hospital Mississauga, ON Canada

**Keywords:** secure messaging, children with medical complexity, patient-physician relationship, care coordination, partnership, email communication, online information, children, pediatrics, caregiver, information sharing, electronic medical record

## Abstract

**Background:**

The Connecting2gether (C2) platform is a web and mobile–based information-sharing tool that aims to improve care for children with medical complexity and their families. A key feature of C2 is secure messaging, which enables parental caregivers (PCs) to communicate with their child’s care team members (CTMs) in a timely manner.

**Objective:**

The objectives of this study were to (1) evaluate the use of a secure messaging system, (2) examine and compare the content of messages to email and phone calls, and (3) explore PCs’ and CTMs’ perceptions and experiences using secure messaging as a method of communication.

**Methods:**

This is a substudy of a larger feasibility evaluation of the C2 platform. PCs of children with medical complexity were recruited from a tertiary-level complex care program to use the C2 platform for 6 months. PCs could invite CTMs involved in their child’s care to register on the platform. Messages were extracted from C2, and phone and email data were extracted from electronic medical records. Quantitative data from the use of C2 were analyzed using descriptive statistics. Messaging content codes were iteratively developed through a review of the C2 messages and phone and email communication. Semistructured interviews were completed with PCs and CTMs. Communication and interview data were analyzed using thematic analysis.

**Results:**

A total of 36 PCs and 66 CTMs registered on the C2 platform. A total of 1861 messages were sent on C2, with PCs and nurse practitioners sending a median of 30 and 74 messages, respectively. Of all the C2 messages, 85.45% (1257/1471) were responded to within 24 hours. Email and phone calls focused primarily on clinical concerns and medications, whereas C2 messaging focused more on parent education, proactive check-ins, and nonmedical aspects of the child’s life. Four themes emerged from the platform user interviews related to C2 messaging: (1) connection to the care team, (2) efficient communication, (3) clinical uses of secure messaging, and (4) barriers to use.

**Conclusions:**

Overall, our study provides valuable insight into the benefits of secure messaging in the care of children with medical complexity. Secure messaging provided the opportunity for continued family teaching, proactive check-ins from health care providers, and casual conversations about family and child life, which contributed to PCs feeling an improved sense of connection with their child’s health care team. Secure messaging can be a beneficial additional communication method to improve communication between PCs and their care team, reducing the associated burden of care coordination and ultimately enhancing the experience of care delivery. Future directions include the evaluation of secure messaging when integrated into electronic medical records, as this has the potential to work well with CTM workflow, reduce redundancy, and allow for new features of secure messaging.

## Introduction

Children with medical complexity are characterized by medical fragility and chronic conditions, and they often require the involvement of multiple specialists across multiple care settings and medical technologies to support activities of daily living [1]. Parental caregivers (PCs) of children with medical complexity often bear the complex burden of coordinating their child’s care, performing tasks such as liaising with multiple care team members (CTMs), coordinating appointments, and merging medical recommendations [2,3]. Some key principles for improving care coordination for children with medical complexity have been suggested, including shared goals, mutual respect, and real-time communication [4].

PCs and CTMs of children with medical complexity have noted several barriers that impede their ability to communicate promptly with members of the child’s health care team, subsequently leading to increased care coordination requirements [5,6]. One such barrier is organizational policies that limit the use of email due to security concerns and promote the use of telephone communication and faxing [6]. A solution to improve these communication challenges and ensuing negative impacts on care coordination is the implementation of real-time communication including email, texting, or secure messaging [6]. Real-time messaging can offer children with medical complexity and their families additional support with the many moving parts of the child’s care, especially outside of medical appointments or admissions to hospitals. Key workers involved in the care of children with medical complexity aim to provide individualized information, support, and direction to families through comprehensive knowledge of a child’s condition and assist with coordination, communication, and follow-through with plans of care [7,8]. As described by the Complex Care for Kids Ontario program standard [9], nurse practitioners (NPs) often play the role of the key clinical worker and are needed as the lead for care coordination for a child with medical complexity. We suggest that real-time communication with key workers for children with medical complexity and their families could enable streamlined care provision, efficient utilization of resources, and improved patient-centered care.

The use of real-time communication methods has been explored in several different pediatric populations and populations of adult patients that require caregivers [10-15]. For instance, telephone-based messaging interventions for caregivers of people with dementia have demonstrated positive impacts on the quality of life for patients, reduced caregiver burden, and reduced emergency department visits [15]. Notably, in pediatric populations, PCs of children with 1 chronic illness (eg, cystic fibrosis, diabetes mellitus, juvenile arthritis, and sickle cell disease) have reported that real-time communication methods helped to reduce barriers in communication, facilitated early intervention in screening, aided referral for treatments, and allowed for the formation of an ongoing relationship with health care providers (HCPs) that was not previously possible [14,16]. However, to our knowledge, there are no studies that examine the use of real-time communication in the care of children with multiple or complex chronic conditions. Therefore, the objectives of this study were 3-fold: (1) to evaluate and compare the use of a real-time secure messaging system by CTMs of children with medical complexity, (2) to examine and compare the content of the messages to that of emails and phone calls, and (3) to explore PCs and CTMs’ perceptions and experiences using secure messaging as a method of communication.

## Methods

### Study Design

This mixed methods evaluation of a web and mobile–based patient-facing platform used messaging, phone call, and email data as well as semistructured interviews to investigate the experiences of PCs and CTMs of children with medical complexity when using a secure messaging system. A mixed methods evaluation enabled us to assess the objective usage of the platform among both CTMs and PCs. It also helped us understand the subjective experiences of users and how they were able to use and incorporate secure messaging into the care of children with medical complexity. This study is a component of a larger study investigating the feasibility of a web and mobile–based patient-facing platform to improve communication and care coordination for children with medical complexity [6].

### Platform Overview

Connecting2gether (C2) is a web and mobile–based patient-facing platform developed for children with medical complexity that supports various functions, including secure messaging, health tracking, educational resources, and a shared medical summary. This platform was developed by using adult literature on secure messaging, referring to caregiver models, and customizing features of the online platform to the health care needs of children with medical complexity. The platform was accessible through desktop, tablet, and mobile devices. The secure messaging feature enabled one-to-one communication through an instant messaging interface. PCs could use secure messaging with CTMs that they invited to C2. All platform users received a push notification to their mobile devices and an email alerting them that they had received a message on C2. Attachments (ie, pictures, videos, and documents) could also be sent in C2 secure messaging. Messages could be responded to immediately, within seconds after a message was received, or days after the message was received.

### Ethics Approval

Institutional research ethics approval was obtained at The Hospital for Sick Children (SickKids; 1000060804), Royal Victoria Regional Health Centre (RVH; R18-013), and Credit Valley Hospital (CVH; 973) [17]. Informed consent was obtained from all participants, and all methods were carried out in accordance with relevant guidelines and regulations. All study data were deidentified before analysis.

### Participant Recruitment

PCs of children with medical complexity were recruited from Complex Care Programs at SickKids, RVH, and CVH. To be eligible for the Complex Care Program, children must meet at least 1 criterion from each of the following conditions: technology dependence and/or users of high-intensity care (eg, mechanical ventilator, constant medical/nursing supervision), fragility (eg, severe/life-threatening condition, an intercurrent illness causing immediate serious health risk), chronicity (condition expected to last at least 6 more months or life expectancy less than 6 months), and complexity (involvement of at least 5 health care practitioners/teams at 3 different locations or family circumstances that impede their ability to provide day-to-day care of decision-making for a child with medical complexity) [18]. Children with medical complexity were also between 0 and 18 years of age at the time of study initiation. Purposive sampling guided parental participant selection to ensure diversity in role, communication experience, age, ethnicity, and location [19,20].

PCs were eligible to participate if they were English-speaking, had access to the internet and a computer, and were the primary caregiver of a child with medical complexity. CTMs were approached prior to recruitment to ensure it was an appropriate time to engage in research for the families (eg, hospitalization, end-of-life, or PC physical/mental health concerns).

In this study, “NPs” refers to the nurse practitioners of children with medical complexity in the Complex Care Program, and “HCPs” refers to other hospital and community–based health care providers. CTMs comprise both NPs and HCPs together.

Every PC had their assigned Complex Care Program NP on the platform. PCs were also able to invite other members of their child’s care team (eg, CTMs like social workers, patient information coordinators, pediatricians, etc) to use C2. CTMs that registered on C2 were presented with the terms of use of the platform and the study information letter. If interested, they were approached by the study research coordinator (RC) and presented with information about the research study and the opportunity to participate. CTMs that declined to participate in the research study were still able to use C2. PCs and NPs received training before registering on C2 (duration of 30 to 60 minutes), and the training presentation was later made available on C2. In addition, CTMs could set up a disclaimer on C2 if they were away or designate time slots in which they would respond to messages (eg, 8 AM to 4 PM) to aid in setting expectations with PCs.

All research study participants received remuneration for participating in the research study. PCs were given CAD $60 (US $44.59) in gift cards (CAD $20 at baseline and CAD $40 after completing the study), and HCPs that completed the end-of-study questionnaire were entered into a draw for a CAD $100 (US $74.32) gift card. Participants that completed the end-of-study semistructured interview received an additional gift card worth CAD $20 (US $14.86). C2 also had a built-in points system where PCs received a specified number of points when completing a platform activity (ie, accessing educational material). As a usage incentive, PCs received a gift card worth CAD $5 (US $3.72) when they reached predetermined point milestones. NPs also received a CAD $5 (US $3.72) gift card for every 50 messages that they sent through C2.

### Data Collection

#### Quantitative Data

Platform users used C2 for 6 months between September 2019 and June 2020. Secure messaging data were extracted from C2. Electronic medical record (EMR) documentation, including email and phone call data during the study period, was extracted from each patient’s EMR. Phone calls were documented by the HCP or NP with a summary of what was discussed and the participants, and emails were summarized or transcribed verbatim and included the number of people on the email thread. The phone call and email data were inputted into the EMR by the HCP anytime after the actual phone calls were completed and email messages were sent and received. Therefore, the time that the phone call and email data were added was not equivalent to when these were completed.

#### Qualitative Data

Platform users were asked to participate in a semistructured interview at the end of the study period. The research team purposefully sampled platform users to include those with high and low platform usage. Informed consent was obtained, and 2 members of the research team (authors CM and MB) led interviews over the phone or via online video conference. Semistructured interview guides explored platform experiences using C2, with specific questions surrounding each platform feature. The qualitative data used in this study included all questions related to the secure messaging function (Multimedia Appendix 1), such as: Can you tell me about your experience using C2 for communication? How did the messaging system compare to your usual methods for communicating with your child’s care team? Were there any barriers to using secure messaging?

### Data Analysis

#### Quantitative Data

All secure messaging, email, and phone call data were deidentified and analyzed using descriptive statistics. “Messaging response” was defined as a secure message that required a response and received a response. The messaging response was categorized subjectively by a single reviewer (author CP) by identifying the messages that required a response (eg, questions) and whether they received a response.

The time and date that each secure message was sent were categorized as evening, weekend, or weekday. Weekday messages were sent between 9 AM EST and 5 PM EST from Monday to Friday. Evening messages were sent after 5 PM EST or before 9 AM EST from Monday to Friday, and weekend messages were sent anytime on Saturday or Sunday.

Content analysis methods and inductive category development [21] were used to analyze the content of secure messages on C2, email messages, and phone calls. First, team members performed an initial review of all secure messaging conversations, email messages, and phone call communication. This led to the development of codes (by authors CP and SM) that were used to categorize each message, for example, medication-related messages, appointment-related messages, inpatient hospital stay–related messages, or messages related to the C2 platform. Following this, discussions took place between members of the research team (authors CP, SM, MB, and JO), and codes were grouped together based on subjective similarities defined by the research team. For instance, appointment and inpatient hospital stay messages were combined under the code “clinical encounter.” A finalized coding tree was developed after analysis of the terms and discussion of disagreements, which led to a total of 9 codes (Multimedia Appendix 2). Disagreements in codes were resolved through discussions between the research team members (authors CP, SM, MB, and JO).

#### Qualitative Data

Semistructured interviews were audio recorded and transcribed verbatim. Transcribed data were managed using NVivo 12 software (QSR International) [22]. We did not have any preconceived themes prior to starting data analysis; instead, themes were developed through an inductive process as a research team. Thematic analysis was used to analyze all interview data, which was completed by 3 team members (authors CP, MB, and CM). Thematic analysis began following the first interview. Braun and Clark’s [23] 6 steps of thematic analysis were adapted. First, the analysis process began with familiarization of the data related to secure messaging, where authors CM and MB transcribed the interview verbatim, read the transcripts, and reread the transcripts while listening to the audio recordings. Following this, through inductive coding, author CP generated initial codes by using an iterative process of reviewing all interview data and discussing potential codes that could be applied to the interview data for both PCs and CTMs with other authors (CM, MB, and JO). For example, the initial code of “fast responses” was deducted from quotes discussing the speed of responses on secure messaging. CP then grouped the codes into potential themes and reviewed the themes using constant comparative analysis by comparing themes across and within participant groups. One example of this was the combination of the initial codes of “informal and simple communication” and “fast responses” into the higher-level theme of “efficient communication.” Three research team members (authors CP, MB, and CM) worked together to refine, define, and name the final 4 themes.

## Results

### Overview

A total of 36 PCs of children with medical complexity registered on C2. Other platform members in the study included 7 NPs and 59 HCPs, 35% (n=21) of whom were pediatricians.

### Quantitative Evaluation of Secure Messaging

During the study period, the RC sent 1818 secure messages to all platform users. PCs sent 1133 (median 30; IQR 13-46) messages, NPs sent 615 (median 74; IQR 37-119.5) messages, and HCPs sent 113 (median 2; IQR 1-4.5) messages. Moreover, 85.45% (1257/1471) of secure messages sent during the study period were responded to within 24 hours, and 92.45% (1360/1471) within 72 hours. PCs replied fastest to HCPs and slowest to the RC. The RC had the fastest response times (16 minutes) compared to all other platform users (Table 1).

**Table 1:** Response time summary by type of platform user.

Platform user type	Median response time (range) (hh:mm)	Response time <24 hours, n (%)	Response time <72 hours, n (%)
PC^a^	0:36 (0:00-547:18)	635 (85.1)	691 (92.6)
RC^b^	0:16 (0:00-304:49)	342 (93.4)	358 (97.8)
NP^c^	2:36 (0:00-290:06)	209 (74.6)	238 (85)
HCP^d^	0:56 (0:01-330:29)	64 (87.6)	67 (91.8)

^a^PC: parental caregiver.

^b^RC: research coordinator.

^c^NP: nurse practitioner.

^d^HCP: health care provider.

The messaging response was 84.53% (1295/1532) for all secure messages over the study period. Most secure messages from PCs (261/280, 93.2%) that required a response from the NP received a response. In messages from PCs to HCPs that required a response, 73% (45/62) received a response.

The majority (2987/3679, 81.19%) of secure messages sent on C2 were sent on weekdays, while 17.09% (n=629) of all messages were sent during evenings, and 1.71% (n=63) were sent on weekends.

**Figure 1:**
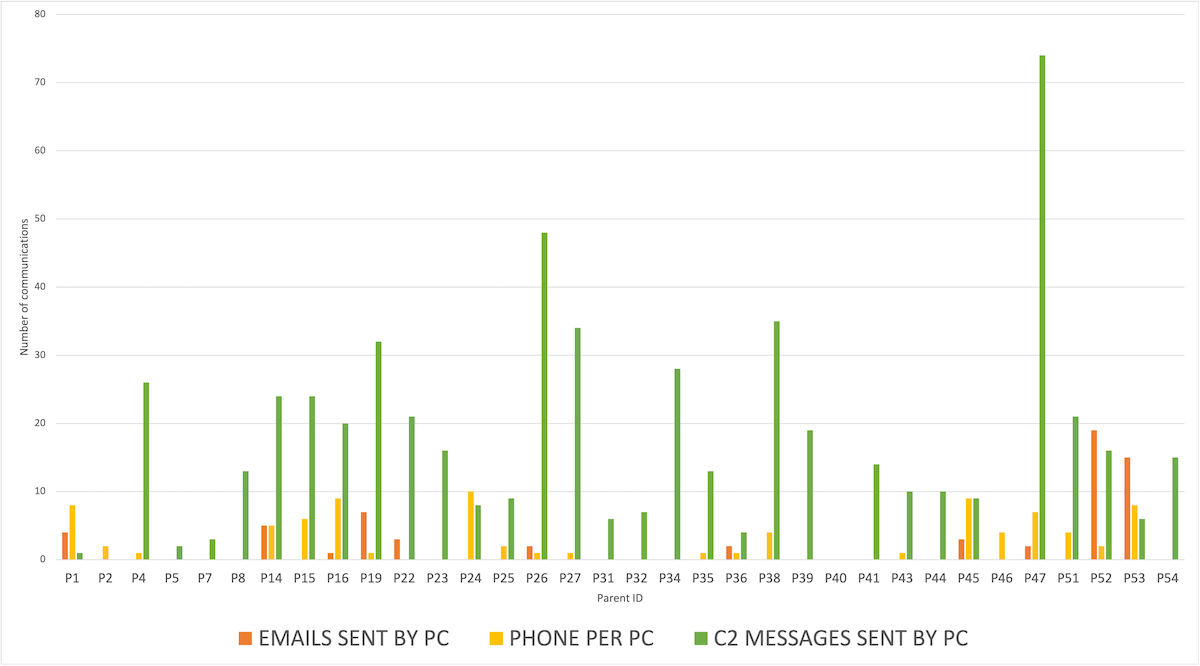
Comparison of the number of Connecting2gether (C2) secure messages, emails, and phone calls by parental caregivers (PCs) to their child’s nurse practitioners (NPs) and health care providers (HCPs) during the study period.

Among the PCs who had phone calls with their care team during the study period (21/36, 58%), there were, on average, 4 (range 1-10) phone calls with their care team (Figure 1). PCs that sent emails to their care team during the study period sent an average of 5 (range 1-19) emails. On average, PCs sent 17 secure messages to both NPs and HCPs on C2.

Due to technical limitations of the platform, conversations on C2 could only include 2 people (ie, the parent and 1 other user). Email allowed individuals to have any number of people within each email thread. Accordingly, 73% (100/137) of the emails contained 2 participants, 19% (n=26) contained 3, 5% (n=7) contained 4, and 3% (n=4) contained 5 participants.

### Qualitative Evaluation of Secure Messaging

#### Content Analysis and Comparison for Secure Messaging, Phone Calls, and Emails

Approximately one-third (1126/3679, 30.60%) of the secure messages on C2 were coded as “C2,” indicating that these messages were related to how to use C2 and its features. These messages were unique to secure messaging on C2 and therefore are not included in Multimedia Appendix 3. The 2 most frequent codes across all 3 forms of communication (secure messaging, email, and phone) were “clinical concern and encounter” and “medications and medical equipment” (Multimedia Appendix 3). There was a higher frequency of “education and resources,” “child and PC life, nonclinical,” and “check-in” codes for secure messaging compared to email and phone communication.

#### Themes From Semistructured Interviews

Four themes emerged from the platform user interview data related to secure messaging on C2: (1) connection to the care team, (2) efficient communication, (3) clinical uses of secure messaging, and (4) barriers to use (Table 2). The first theme (connection to the care team) focuses on how secure messaging impacted access to CTMs and the personal relationships and connections PCs developed with their CTMs. For instance, PCs discussed how beneficial it was to be able to communicate directly with their care team. The second theme (efficient communication) focuses on the convenience and ease of using C2, which both CTMs and PCs endorsed. The third theme (clinical use of secure messaging) describes how PCs and CTMs used secure messaging to improve their clinical experiences. For example, secure messaging was used to supplement in-person visits, discuss medication changes, and communicate with community-based HCPs. The fourth theme (barriers to use) discusses the limitations of secure messaging, how it could be improved, and why some platform users prefer to use email and phone. Many PCs and CTMs mentioned that the extra steps to log into C2 to read and respond to messages acted as a deterrent and described that at times they were unsure of when to use each communication platform. It is important to note, however, that in interviews with CTMs, none expressed that there was an increase in their workload due to the volume of messages being sent.

**Table 2:** Quotes reflecting key themes related to secure messaging on the C2^a^ platform.

Themes	PC^b^ quotes	CTM^c^ quotes
Connection to the care team	“Over time it became a partnership between the care team and [us]. There’s an invitation there to suggest treatments or alternatives. We [were] able to be taken seriously and to start this cooperation in the care of our daughter.” PC#8 “I feel heard, and more connected because of the [NP’sd] quick response. We feel that we’re genuinely being taken care of by her, and that she’s concerned, not just about my son and the medical side, but just our family’s wellbeing in general. That definitely came through, because of [C2].” PC#23	“[With messaging on C2], it feels less formal than email so you can just send like a quick, one or two lines to the family.” CTM#3 “[With] email, the amount of detail [parents] go into might be a little bit more. With [C2], they were more direct, [showed] their concerns, and [asked] their questions.” CTM#35
Efficient communication	“It’s easier to pull up previous conversations in a message, as opposed to different emails all over the place. If I kept scrolling up [on C2] then I could see what we had talked about two months ago.” PC#26 “I found [C2 to be] an easy way to send off quick messages. I think it was more friendly or laid-back… Maybe more casual.” PC#22 “I think my favourite [C2 feature] was the messenger. To be able to contact the pediatrician directly, instead of trying to call an office, and then trying to book a time for us to be able to chat with him.” PC#54	“I would say [email and C2] are similar. And perhaps [C2] was collated, better. [Emails] can get buried with other emails but [on C2 conversations are] specific to that patient so [there is] a bit of a trail.” CTM#16 “[With] email the amount of detail they go into might be a little bit more. With [C2] I felt like [PCs] were able to be more direct, show their concerns, and ask their questions.” CTM#35 “I found that it was much quicker to get a hold of them [via C2], rather than email or calling [PCs].” CTM#27
Clinical uses of secure messaging	“I think [C2 is] a great supplement. For example, our Complex Care meetings will be twice a year. Stuff happens in the six months in between and that is where [C2] was very helpful.” PC#8 “[The community-based social worker and I] had a lot of back-and-forth based on accessibility equipment that we were having ordered and measured. [We were] able to communicate with her when she had a quick question, or if we were relaying information to her, as well as ask questions and prepare things between visits. [C2] made that piece of it easier for us.” PC#19 “It was really nice that [our NP] would [message] and say, “Hey, just checking in, how’s [son] doing?”. Had I not had this platform, I wouldn’t have told her that [for example] he’s got a cough. We would deal with it at home and if need be, we would go to the Emergency in our town. She’s more up-to-date with what was going on with him.” PC#26	“If it’s not something that’s a new onset of a certain symptom or a certain issue, then the messaging platform is fine, especially dealing with ongoing issues. When something new has happened, and you need more detail, then I can see the email or phone might be more useful.” CTM#35 “I mainly used [C2] if there was a follow-up from an [previous] issue, as a check-in but also, if they had an upcoming visit, reaching out to them just to see if there’s anything in particular that they wanted to discuss at the visit.” CTM#03 “I think [C2] allows continuity between [visits] for things that we’re trying to work on. I think this [C2] messaging] would allow [for] communication between the clinical team and the family [between appointments]. It would mean that you’re using the time between appointments.” CTM#40
Barriers to use	“The helpfulness [of C2] was limited in this study period primarily because the health care providers on my son’s [care] team didn’t accept the invitations to participate.” PC#36 “If there was a way [on C2] to, communicate with all three [HCPs] at the same time, to get the same message to all of them, that would be a benefit” PC#47 “I normally use email because it’s the most convenient. Requires me to take the fewest extra steps in order to send the message. I am usually [already] logged into my email anyway.” PC#53	“It would be nice if [C2] were, linked to [the EMRe]. It’s hard to keep track of all of the different ways that people can try to get in touch with you and it’s easy to miss [a message] if you don’t respond right away. Adding another system makes me worry that I wouldn’t be able to handle everything.” CTM#42 “My issue with using all three [forms of communication is that parent] wasn’t sure which one was the “right” way to message me. So she wouldn’t consistently use the same [form of communication].” CTM#25

^a^C2: Connecting2gether.

^b^PC: parental caregiver.

^c^CTM: care team member.

^d^NP: nurse practitioner.

^e^EMR: electronic medical record.

## Discussion

### Principal Findings

The use of secure messaging as a means of communication in health care is a relatively novel concept in the pediatric literature. To our knowledge, this is the first mixed methods study investigating the use and perceptions of a secure messaging system housed in a web and mobile–based patient-facing platform for PCs and CTMs of children with medical complexity. Our findings demonstrate that secure messaging was highly used, allowed for diverse topics of conversation, and enhanced the PC-CTM relationship. This paper also demonstrated the utility of secure messaging as a significant collaborative tool between CTMs and parental caregivers, as demonstrated by the large volume of messages exchanged, as an addition to existing forms of communication. In addition to medical information and administrative conversations, secure messaging has surrounded the sharing of educational resources, proactive check-ins initiated by CTMs, and general conversation about the child/family life. Our discussion of this paper will focus on 3 key areas: the clinical utility of secure messaging, the enhancement of the role of the PC with secure messaging, and the workflow and risks of secure messaging.

There is limited pediatric literature to date showing the clinical utility and role of SM; however, there has been noted enthusiasm about the exploration of this topic [11,24,25]. There is mixed evidence in the literature of how secure messaging could be used clinically. Only 1 study with children and adolescents with sickle cell disease found that messaging was used for proactive check-ins and sharing of educational information [14], whereas a study with pediatric surgical patients did not [26]. Considering the current literature, our findings suggest that children with higher care needs and chronic conditions, such as those with medical complexity and those with sickle cell disease, benefit from the use of secure messaging as it allows for proactive check-ins from their CTMs as well as continued education surrounding their medical conditions. For children with medical complexity, there is a need for constant check-ins from CTMs, and secure messaging enables this. In particular, for these children and their families, there is a clinical utility in accessible secure messaging, as it enables prompt clinical response to the high acuity issues we frequently see in this population. Our finding suggesting that secure messaging could be a modality to support parental education is especially salient as previous research has shown that PCs of children with medical complexity often wish to learn more about their child’s condition(s) and how to provide care at home [27,28].

Taken together, we suggest that all the noted benefits of secure messaging can contribute to reducing the burden of care coordination for PCs of children with medical complexity. The four defining characteristics of care coordination include (1) family-centeredness; (2) planned, proactive, and comprehensive focus; (3) promotion of self-care skills and independence; and (4) emphasis on cross-organizational relationships [4]. Indeed, improving the connection and ease of communication between PCs and CTMs increases the opportunity for partnership, family-centered care, and independence, while the finding of proactive check-ins by CTMs aligns well with a planned, proactive, and comprehensive focus.

A barrier to communication between PCs of children with medical complexity and their CTMs is a perceived lack of partnership, as PCs do not feel acknowledged for their expertise and cannot contribute to their child’s care plan [6]. PCs in our study noted that secure messaging allowed them to play a more active role in their child’s care, allowed for more informal conversations with their child’s health care team, and ultimately improved their perceived partnership with their child’s health care team. This finding is supported in adult literature; a study of women with breast cancer demonstrated that the use of an interactive communication tool allowed participants to feel more empowered to participate in their health care and improved their relationship with their health care team [29].

A common concern in the literature surrounding real-time communication and secure messaging is that patients may overwhelm CTMs by sending many messages, significantly increasing their workload [30,31]. In this study, PCs and NPs received training on the appropriate use of C2 prior to registering. Research on the use of patient portals in primary care clinics demonstrates that our efforts to provide clear and structured training addressed any concerns on how to communicate with CTMs appropriately [32]. Our research adds to the literature demonstrating that patients do not overuse secure messaging systems [33]. The findings of this paper dispel the concerns of secure messaging overuse because secure messaging did not add significantly to the workload for CTMs, and it can be constricted to weekday daytime hours.

Both PCs and CTMs noted limitations that affected their use of the secure messaging system on C2. These limitations, including additional log-in steps associated with secure messaging and difficulty identifying which communication method to use, are similar to those reported by Hsiao et al [34], who implemented a secure messaging platform in the care of pediatric patients with respiratory diseases. CTMs in our study felt that having the secure messaging system integrated with their EMR would greatly reduce any barriers to access and the additional log-in steps required. Indeed, previous research has demonstrated a high uptake of secure messaging systems when integrated into a patient portal and EMR [35]. In this study, CTMs were required to both communicate by secure messaging and copy the conversation into the patient’s chart. Secure messaging as a part of the EMR would thus lead to reduced redundancy in separate health information platforms, as all information would be housed in a single system. In addition, if they are separate systems, other people who are not using the separate platform would not have access to the secure messages that are important aspects of their child’s care. The integration of secure messaging into the EMR also enables messaging with patients to become a part of a CTM’s standard workflow and includes the messaging as a permanent record in the EMR [36]. Moreover, having secure messaging as a core function within an existing EMR would enhance health care delivery. Specifically, this would allow the provider to respond to secure messages with access to accurate and real-time information including up-to-date medication lists, upcoming appointment times, and hospitalizations or emergency department encounters. Ultimately, including secure messaging in the EMR blends well into the workflow of CTMs and leads to family-centered care, wherein all care providers and PCs have access to the same information. Future research should focus on health-related outcomes (for instance, hospitalizations and emergency department visits) associated with integrating messaging into the EMR, as well as perceptions of HCPs on how integration affected their clinical workflow.

### Limitations

This study has several limitations. First, most of our relatively small PC sample who used secure messaging on the web-based patient-facing platform were highly educated and had high household incomes. Therefore, our results may not be representative of families with lower education, health literacy, or income. However, previous research has shown that economically disadvantaged families still access the internet regularly and are interested in receiving hospital communication through electronic means [12]. Further, the platform was only available in English, and we did not collect PC immigration status. Using secure messaging may present different benefits and challenges to families who are new to Canada and those who do not speak English fluently. There was a possible participation bias, as PCs who chose to use the platform for 6 months and participate in the research study likely already had positive perceptions of and were comfortable using technology such as smartphones, the internet, and computers. However, we attempted to mitigate this bias in the semistructured interviews by recruiting high and low platform users. Finally, this study’s primary focus was on the evaluation of platform usage. Further analysis will include understanding the individual-level characteristics and utilization patterns of secure messaging.

### Conclusions

Our study demonstrates that secure messaging was an effective and additive form of communication for PCs of children with medical complexity and their CTMs that had a high uptake among users. Through a web and mobile–based patient-facing platform, secure messaging provided the opportunity for continued patient education, proactive check-ins from CTMs, and casual conversations about family and child life, all of which contributed to PCs feeling improved connections with their child’s health care team. Therefore, we suggest that secure messaging can improve communication between PCs of children with medical complexity and their care team to reduce the burden of care coordination and ultimately improve the experience of care delivery. Future research should focus on a more rigorous evaluation of secure messaging in the care of children with medical complexity and how it can affect health-related outcomes, especially when integrated into the EMR.

## Appendix

Multimedia Appendix 1 Semistructured interview questions for parental caregivers (PCs) and care team members (CTMs).

Multimedia Appendix 2 Code tree for secure message, phone call, and email communications.

Multimedia Appendix 3 Content analysis of (A) secure messaging on Connecting2gether (C2), (B) email communications, and (C) phone calls. PC: parental caregiver.

## Abbreviations

C2: Connecting2gether
CTM: care team member
CVH: Credit Valley Hospital
EMR: electronic medical record
HCP: health care provider
NP: nurse practitioner
PC: parental caregiver
RC: research coordinator
RVH: Royal Victoria Regional Health Centre
SickKids: The Hospital for Sick Children

## Data Availability

The data sets generated and analyzed during this study are available from the corresponding author upon reasonable request.
