# Supplementary material for: Evaluation of a Secure Messaging System in the Care of Children With Medical Complexity: Mixed Methods Study
Source: JMIR Form Res. 2023 Feb 23;7:e42881. doi: 10.2196/42881 (PMC9999262; doi:10.2196/42881)
Supplement: Multimedia Appendix 1 [file formative_v7i1e42881_app1.docx]

CTM Secure Messaging Questions:

Can you tell me about your experience using Connecting2gether for communication? Probes: What was communication like with the family? Other care team members? How did the messaging system compare to your usual methods for communicating with the family/care team? Were there any barriers to using the messaging? How did you decide between using different methods (email, phone, messaging)? How did the response time on the platform compare to other methods? How did using the platform compare to in-person interactions?

PC Secure Messaging Questions:

You were able to send messages to anyone within your circle of care on Connecting2gether. Can you tell me about your experience using Connecting2gether for communication? Probes: What was your communication like with your Complex Care Team? SickKids team? Community care team members? Others that you added? How did the messaging system compare to your usual methods for communicating with your child’s care team? Were there any barriers to using the messaging? How did you decide between using different methods (email, phone, messaging)? How did the response time on the platform compare to other methods? How did using the platform compare to in-person interactions?
