# Supplementary material for: Evaluation of a Secure Messaging System in the Care of Children With Medical Complexity: Mixed Methods Study
Source: JMIR Form Res. 2023 Feb 23;7:e42881. doi: 10.2196/42881 (PMC9999262; doi:10.2196/42881)
Supplement: Multimedia Appendix 2 [file formative_v7i1e42881_app2.docx]

| **CODE** | **DEFINITION** |
| --- | --- |
| C2 PLATFORM | Messages related to the actual functioning of the platform (including questions and concerns about the platform) and messages referencing the features of the platform |
| MEDICATIONS AND MEDICAL EQUIPMENT | Messages related to the CMC’s medications, medical equipment, or medical technology |
| CLINICAL CONCERN AND ENCOUNTER | Messages related to the CMC’s clinical conditions (e.g. concerns, new symptoms, goals of care) and related to an message relating to an admission or emergency department visit |
| ADMINISTRATIVE | Messages related to administrative tasks for patient care (e.g. referral, scheduling, and provision of letters or forms) and research studies |
| DIAGNOSTIC TESTING AND IMAGING | Messages related to the ordering, outcomes and communication of testing and imaging |
| CHECK-INS | Check-in messages that are initiated by a healthcare provider before a clinic visit to identify the items the PC wants to discuss during that visit or check-in messages that are unrelated to a clinic visit to see how the patient is doing in general or with respect to a specific clinical issue |
| CHILD AND PC LIFE, NON-CLINICAL | Messages and conversation outside of the patient’s clinical care, including aspects of their quality of life, their life outside of their medical care, communication, etc. |
| EDUCATION AND RESOURCES | Messaging containing health-related education and/or resources for families |
| CARE COORDINATION | Messages related to the coordination of care for patients across and within facilities |
