# Supplementary figures and images for: Evaluation of a Secure Messaging System in the Care of Children With Medical Complexity: Mixed Methods Study
Source: JMIR Form Res. 2023 Feb 23;7:e42881. doi: 10.2196/42881 (PMC9999262; doi:10.2196/42881)

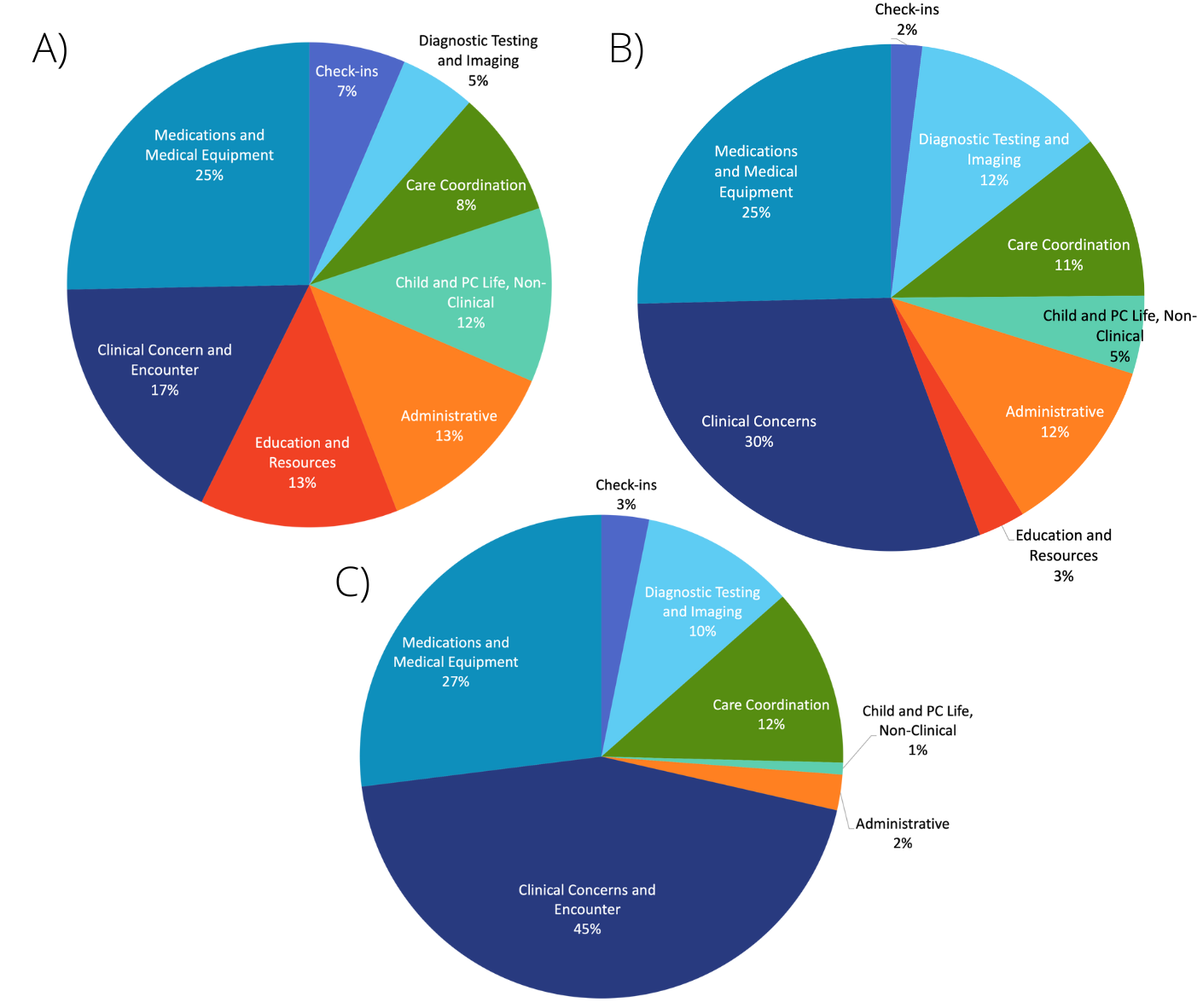

Supplement: Multimedia Appendix 3 [file formative_v7i1e42881_app3.png]
